# Repeated diagnostic ultrasound exposure modifies the structural properties of CA1 dendrites and alters the hippocampal transcriptome

**DOI:** 10.1038/s41598-024-62621-y

**Published:** 2024-05-22

**Authors:** Zsuzsanna Winkler-Ferenczi, Bence Pelyvas, Marianna Nagy, Maria Marosi, Monika Beresova, Rita Varga, Janos Bencze, Peter Szucs, Ervin Berenyi, Angelika Englohner, Zoltan Meszar, Tamas Papp

**Affiliations:** 1 Department of Medical Imaging, Faculty of Medicine, University of Debrecen, Debrecen, Hungary 4032; 2 Department of Anatomy, Histology and Embryology, Faculty of Medicine, University of Debrecen, Debrecen, Hungary 4032; 3 HUN-REN-DE Neuroscience Research Group, Debrecen, Hungary

**Keywords:** Developmental biology, Neuroscience

## Abstract

The development of neurons is regulated by several spatiotemporally changing factors, which are crucial to give the ability of neurons to form functional networks. While external physical stimuli may impact the early developmental stages of neurons, the medium and long-term consequences of these influences have yet to be thoroughly examined. Using an animal model, this study focuses on the morphological and transcriptome changes of the hippocampus that may occur as a consequence of fetal ultrasound examination. We selectively labeled CA1 neurons of the hippocampus with in-utero electroporation to analyze their morphological features. Furthermore, certain samples also went through RNA sequencing after repetitive ultrasound exposure. US exposure significantly changed several morphological properties of the basal dendritic tree. A notable increase was also observed in the density of spines on the basal dendrites, accompanied by various alterations in individual spine morphology. Transcriptome analysis revealed several up or downregulated genes, which may explain the molecular background of these alterations. Our results suggest that US-derived changes in the dendritic trees of CA1 pyramidal cells might be connected to modification of the transcriptome of the hippocampus and may lead to an increased dendritic input.

## Introduction

Ultrasound (US) is a mechanical pressure wave with a frequency exceeding 20 kHz, the upper limit of human hearing; in medical imaging, the applied frequency range is 1–15 MHz. During fetal ultrasound, the examination can be performed with the application of convex abdominal or vaginal transducers (the frequency range is 3–12 MHz) with mechanical index (MI) < 1 and thermal index (TI) < 1^1,2^. MI is an indication of an ultrasound beam's ability to cause cavitation-related bioeffects, TI indicates the elevation of temperature in the soft tissue^1^. Even below these MI and TI criteria, the US exposure acts on the central nervous system at a cellular level by altering neuronal morphology, resulting in the elaboration of dendritic trees in vitro^3^ and in vivo^4,5^. These effects could be more profound with the use of repetitive ultrasound exposure that significantly increases the mean length of neurite outgrowth and leads to the proliferation of neuroblasts^6,7^. At the subcellular level, the effect of US on neurodevelopment is particularly mediated by the activation of mechanosensitive ion channels (MSCs), such as TRPC4 and TRPA1 (US frequency range 1–7 MHz), causing BDNF and c-fos elevation, which are markers of neural activity^4,8,9^. BDNF is known to increase dendritic spine density in apical dendrites and modify the spine morphology of CA1 pyramidal neurons^10,11^. Nevertheless, synaptic activity (without specific TRPC4 or TRPA1 activation) itself affects the size, shape, and maintenance of spines resulting in constant changes even in the adult brain^12,13^. Following this recent studies claim that the spine density is reduced in major neurological diseases (e.g. Parkinson’s and Huntington’s diseases, schizophrenia, Alzheimer’s disease, and major depression), some of which have neurodevelopmental determination^14–16^. In a previous study^4^, we found that a single exposure to obstetric ultrasound has minor but long-lasting effects on the dendritic arborization of layer V pyramidal cells in the retrosplenial cortex. Specifically, the exposure increased the quantity of basal dendrites. In our recent experiment, we looked into the potential effects of repeated ultrasound exposure on the spine density and morphology of pyramidal cells in the CA1 region. Our results show that ultrasound exposure significantly increases the spine density at the basal dendrites of hippocampal pyramidal cells. This increase in spine density could potentially expand the receptive field and improve neuronal activity. Furthermore, our findings indicate that exposure to ultrasound also leads to a significant increase in both the average segment length and dendritic diameter within the basal dendritic tree of CA1 neurons.

## Results

### Hippocampal transcriptome analysis

**Figure 1:**
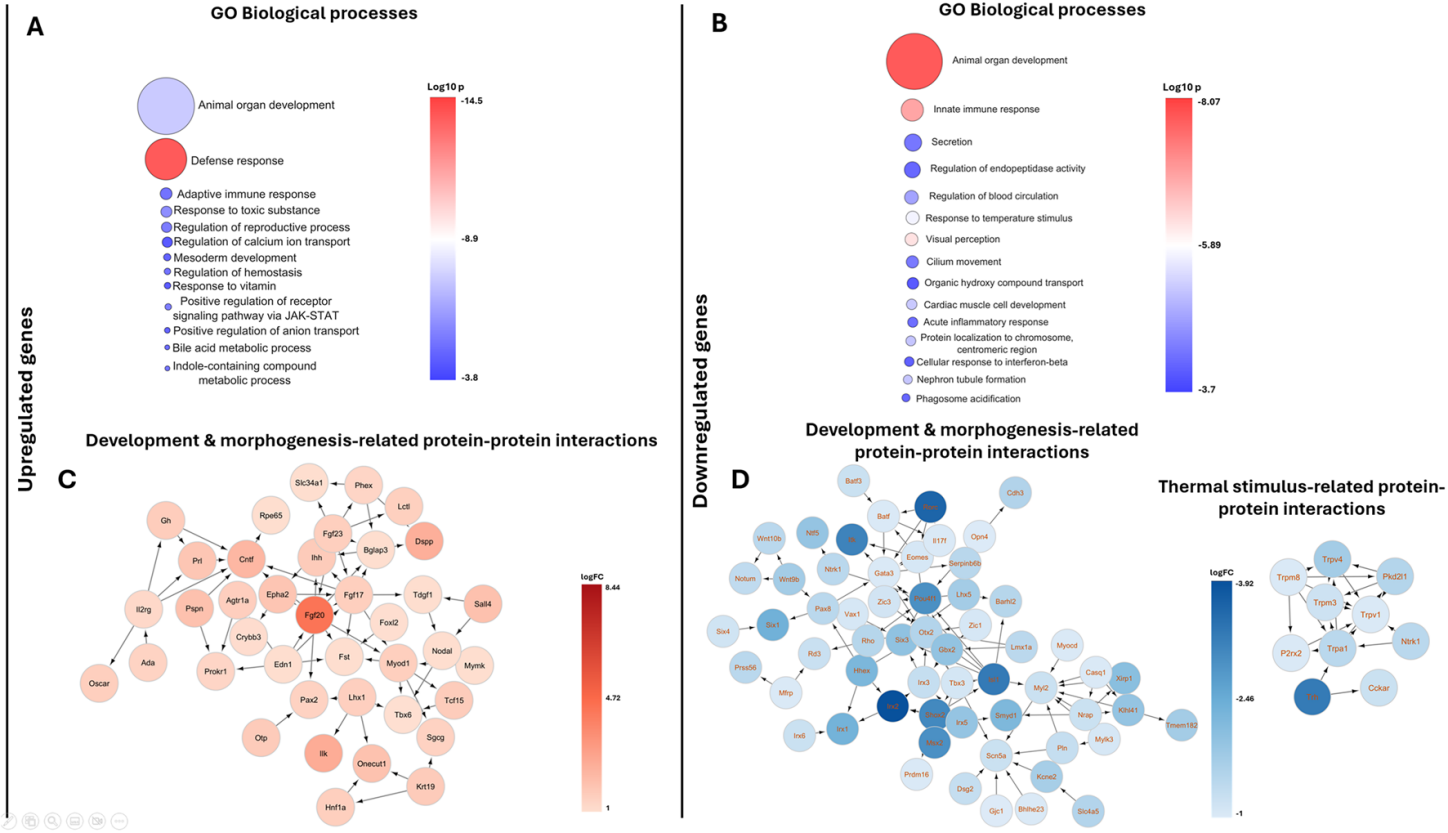
Comprehensive RNAseq analysis and GO biological processes. The figure is divided into four panels, each representing different aspects of the RNAseq differential expression (DE) analysis. The upper panels generated by the EnrichmentMap (ver 3.3.6) show the GO Biological Processes involved in upregulated (**A**) and downregulated (**B**) sequences with a cut-off of less than twofold. The size of each bubble corresponds to the number of genes involved in each process, while the color indicates the p-value (log10 value). Lower panels show putative Protein–protein interactions related to FGF20 mediated morphogenesis (**C**) and developmental-related down regulated proteins involved in neurogenesis (**D**). Lower panel (**D**) also illustrates proteins response to thermal stimuli revealed by the Functional enrichment module of the Cytoscape (ver 3.10.1)^39^.

Differential expression (DE) analysis of our bulk RNAseq samples (taken from 5 times the US treated vs control), the results of the analysis are presented in Suppl. 1. The highest upregulation was seen related to the *Gdf1* gene (FC: 347.38, logFC: 8.44), while the *Ccnd3-ps* showed the strongest downregulation (FC: − 10.45 logFC: 3.38). Using EnrichmentMap (ver 3.3.6), our analysis revealed several putative GO Biological functions in the up-and down-regulated datasets with the highest impact on the organogenesis-related processes^17^. Since we were analyzing pools of the hippocampus, we focused only on the highly up- or downregulated genes, which are interested in the signal transduction of US and dendrite or spine development, we found multiple pathway modulation related to the treatment, not a well described signaling pathway. Among them, we found that the expression of the *Fgf20-mediated* morphogenesis, *Ilk,* and other morphogenetic molecules like *Mir-546*, and *Eps8l3* was higher in the US-treated hippocampus. On the other hand, we found that several non selective cation channels which may take a part during signal transduction went through downregulation (Fig. 1).

### Ultrasound stimulus increases the spine density of basal dendrites and modifies the morphology of the spines on hippocampal pyramidal cells

**Figure 2:**
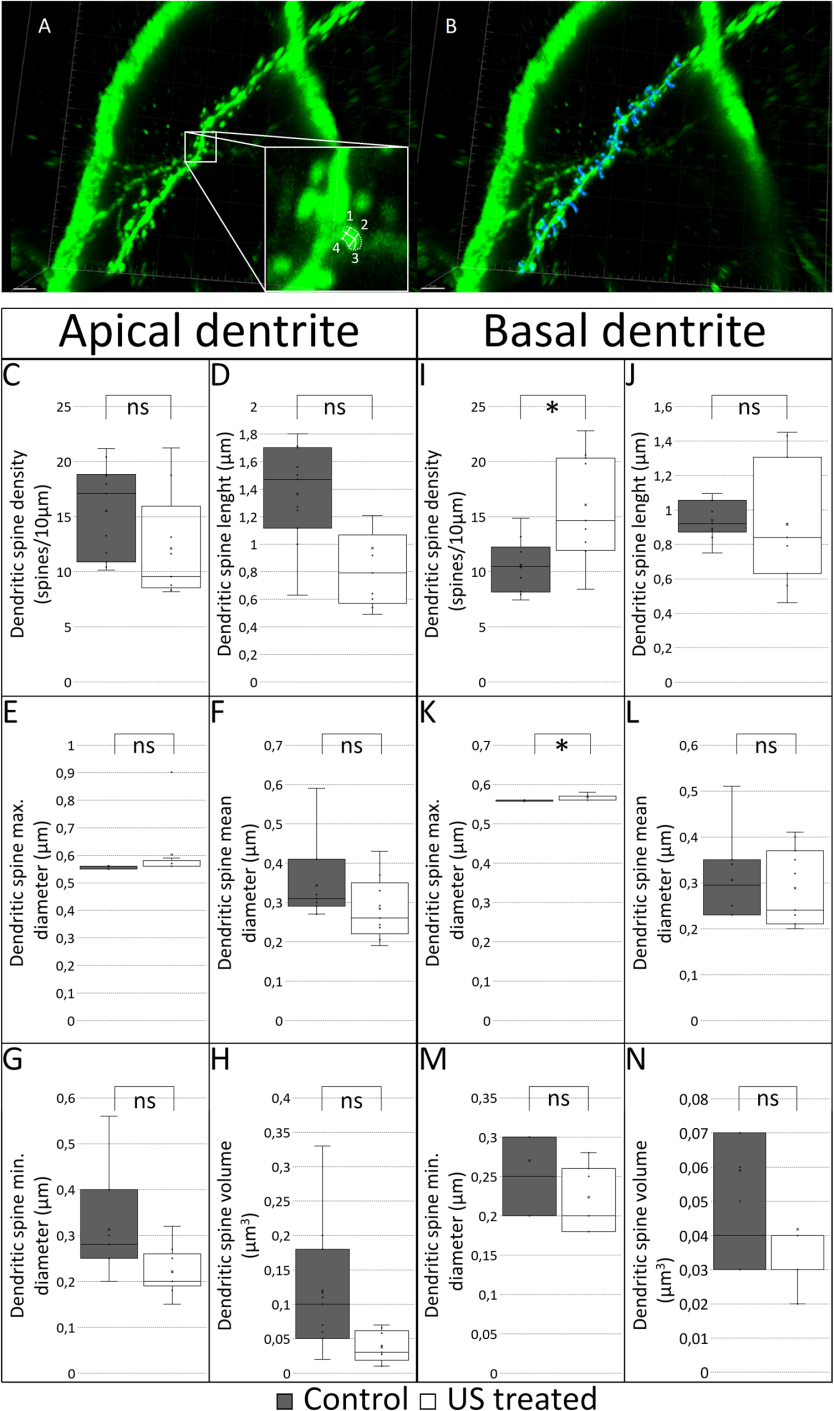
The semiautomatic analysis of dendritic spines with Imaris on the secondary dendrites of CA1 neurons. The (**A**) panel shows a representative immunofluorescence image (in the white box the number 1 labels the min. spine diameter, 2 the max. spine diameter, 3 the spine length, and 4 the spine volume); in the (**B**) panel the software labeled with blue the detected spines. Scale bar: 3 µm. Quantification of the apical dendritic spine characteristics (control versus US treated group). Spine density (**C** panel, p = 0.11, control 15.5069 ± 1.2840, US treated 12.1175 ± 1.5933). Spine length (**D** panel, p = 0.10, control 1.3638 ± 0.1064, US treated 0.9705 ± 0.2204). Spine maximum diameter (**E** panel, p = 0.23, control 0.5600 ± 0.0043, US treated 0.6022 ± 0.0375). Spine mean diameter (**F** panel, p = 0.15, control 0.3436 ± 0.0286, US treated 0.2840 ± 0.0265). Spine minimum diameter (**G** panel p = 0.026, control 0.3127 ± 0.0311, US treated 0.2211 ± 0.0171). Spine volume (**H** panel, p = 0.019, control 0.1173 ± 0.0270, US treated 0.0376 ± 0.0075). Significant difference labeled by: *p ≤ 0.017) According to the MANOVA test, we found a significant difference; p: 0.02. Quantification of the basal dendritic spine characteristics (control versus US treated group). Spine density (**I** panel, p = 0.004, control 10.5728 ± 0.7592, US treated 16.0689 ± 1.4284). Spine length (**J** panel, p = 0.83, control 0.9396 ± 0.0343, US treated 0.9150 ± 0.1023). Spine maximum diameter (**K** panel, p = 0.014, control 0.5580 ± 0.0013, US treated 0.5673 ± 0.0030). Spine mean diameter (**L** panel, p = 0.64, control 0.3060 ± 0.0287, US treated 0.2882 ± 0.0249). Spine minimum diameter (**M** panel, p = 0.15, control 0.2700 ± 0.0300, US treated 0.2236 ± 0.0122). Spine volume (**N** panel, p = 0.32, control 0.0590 ± 0.0155, US treated 0.0418 ± 0.0080). Significant difference labeled by: *p ≤ 0.017) According to the MANOVA test, we did not find a significant difference; p: 0.1.

To show the effects of repeated US on the properties of dendritic spines, semiautomatic analysis of hippocampal pyramidal cells was performed with Imaris (Suppl. 2) and Neurolucida. We analyzed 1227 spines in total (treated samples: 343 basal and 235 apical; control samples: 220 basal and 429 apical) (Fig. 2). In the case of apical dendrites, the minimum diameter and volume were found higher in the control group, while in the case of those animals stimulated five times with US the basal dendritic spine density and the maximum diameter of spines were significantly higher. No change was observed in mean spine diameter and spine length, neither on apical nor on basal dendrites. The raw data of the morphometry is available in Suppl. 3 table.

### Repeated ultrasound exposure changes the morphological characteristics of CA1 neurons

**Figure 3:**
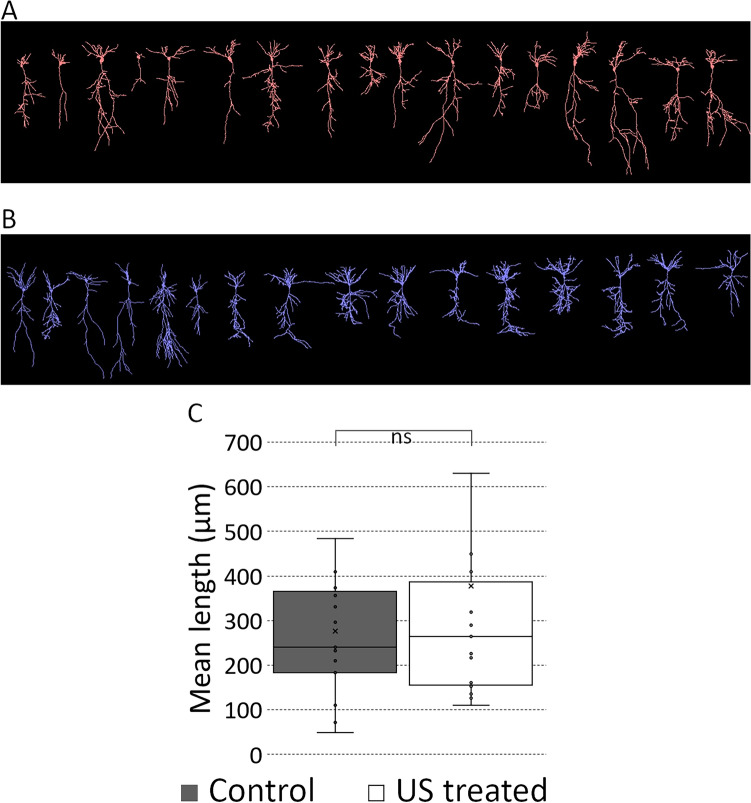
The US treatment not changed the mean dendritic length of CA1 excitatory neurons. (**A**) and (**B**) panels show reconstructions of the control- and US-treated CA1 excitatory neurons. Quantification of the mean dendritic length (**C** panel, p = 0.9, control 276.44 ± 38.78, US treated 376.7 ± 112.53). Between the mean length, we did not found significant. Scale bar: 400 µm.

**Figure 4:**
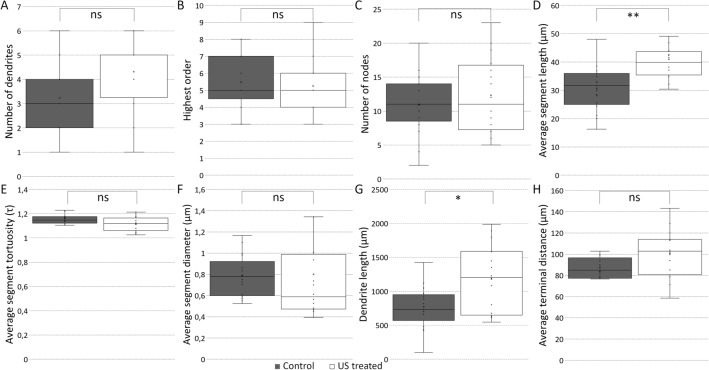
US treatment changed several morphological properties of the basal dendritic tree of CA1 excitatory neurons. Quantification of the basal dendritic tree characteristics (control versus US treated group). Number of dendrites (**A** panel, p = 0.0323, control 3.24 ± 0.32, US treated 4.31 ± 0.26). Highest order (**B** panel, p = 0.6739, control 5.47 ± 0.37, US treated 5.25 ± 0.36). The number of nodes (**C** panel, p = 0.4233, control 10.88 ± 1.08, US treated 12.31 ± 1.39). Average segment length (**D** panel, p = 0.0011, control 30.66 ± 1.89, US treated 40.93 ± 2.21). Average segment tortuosity (**E** panel, p = 0.051, control 1.16 ± 0.02, US treated 1.11 ± 0.02). Average segment diameter (**F** panel, p = 0.2009, control 0.78 ± 0.05, US treated 0.80 ± 0.12). Dendrite length (**G** panel, p = 0.008, control 771.3 ± 74.70, US treated 1188.82 ± 124.07). Average terminal distance (H box; μm) (p = 0.0491, control 83.19 ± 6.40, US treated 100.53 ± 5.53). Significant difference labeled by: **p ≤ 0.01; *p ≤ 0.017. Using the MANOVA test we found a significant difference; p: 0.0009.

**Figure 5:**
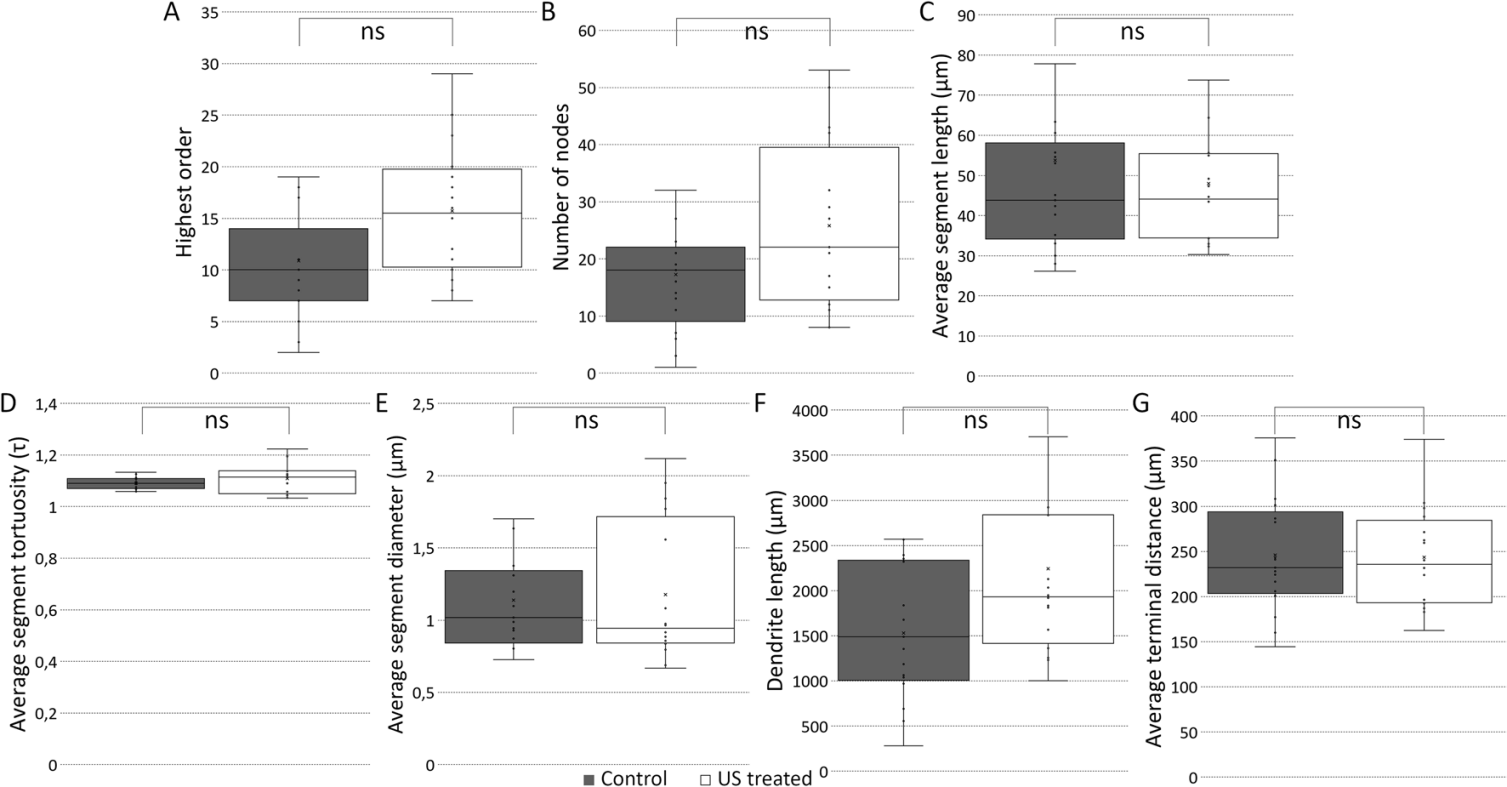
US treatment does not change the apical dendritic tree of CA1 excitatory neurons. Quantification of the apical dendritic tree characteristics (control versus US treated group). Number of dendrites (**A** panel, control 1, US treated 1). Highest order (**B** panel, p = 0.03768, control 10.88 ± 1.53, US treated 15.69 ± 1.60). Number of nodes (**C** panel, p = 0.0701, control 17.24 ± 2.65, US treated 25.75 ± 3.66). Average segment length (**D** panel, p = 1, control 53.72 ± 8.84, US treated 47.97 ± 4.27). Average segment tortuosity (**E** panel, p = 0.2478, control 1.09 ± 0.01, US treated 1.11 ± 0.01). Average segment diameter (**F** panel, p = 0.7594, control 1.14 ± 0.09, US treated 1.18 ± 0.12). Dendrite length (**G** panel, p = 0.05396, control 1530.48 ± 176.52, US treated 2240.96 ± 283.30), Average terminal distance (**H** panel, p = 0.9209, control 245.62 ± 15.55, US treated 243.53 ± 13.87). Significant difference labeled with *p ≤ 0.017. Using the MANOVA test we also did not find a significant difference; p: 0.3523.

According to the morphometric analysis, the mean length of the dendrites did not change (Fig. 3). Confirming our earlier findings^4^, the US exposure elevated the number of basal dendrites but did not alter the number of dendrites on the apical dendritic tree, also in the case of the basal dendrites the dendrite length and segment length showed strong elevation (p ≤ 0.01) as a result of US treatment. The average terminal distance was also significantly elevated in the case of basal dendrites (Fig. 4). The apical dendritic tree properties did not show significant alteration (Fig. 5). With MANOVA test we found significant differences related to the investigated dendritic tree properties between the groups in sum; p: 0.004.

### Morphological analysis of the hippocampal region with micro CT

**Figure 6:**
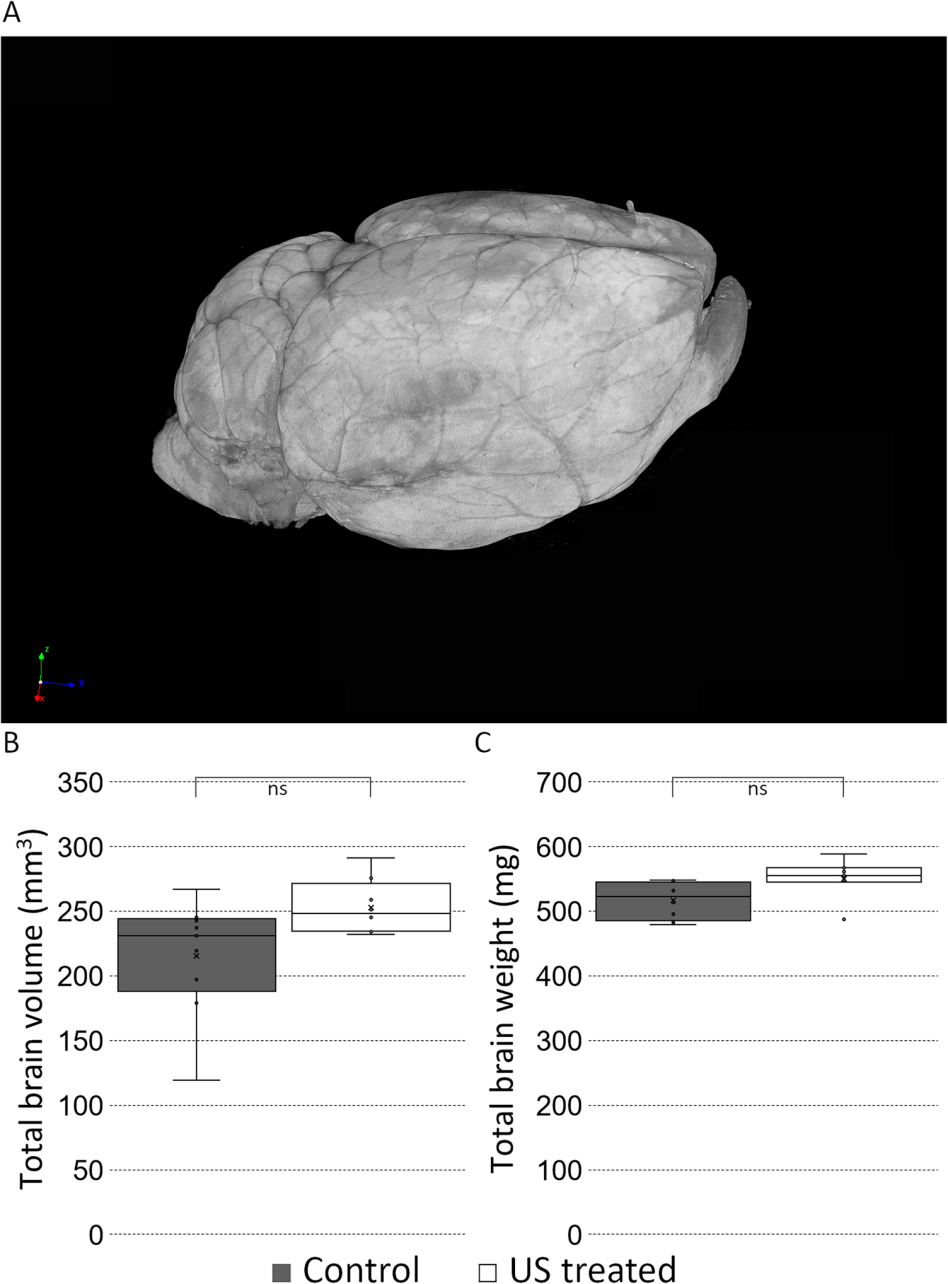
Five times repeated ultrasound exposure did not alter total brain volume and total brain weight. A representative 3D volume-rendered reconstruction of a 1-year-old mouse’s brain (**A** panel). Scale bar: 2 mm. Quantification of the total brain volume (**B** panel, p = 0.06 control 215 ± 14.8997, US treated 253 ± 7.5106); total brain weight (**C** panel, p = 0.04, control: 517 ± 10.0125, US treated 550 ± 11.8290). No significant difference could be detected in total brain volume and total brain weight between the control and the US-treated groups (p ≤ 0.01). MANOVA test did not show a significant difference; p: 0.09.

**Figure 7:**
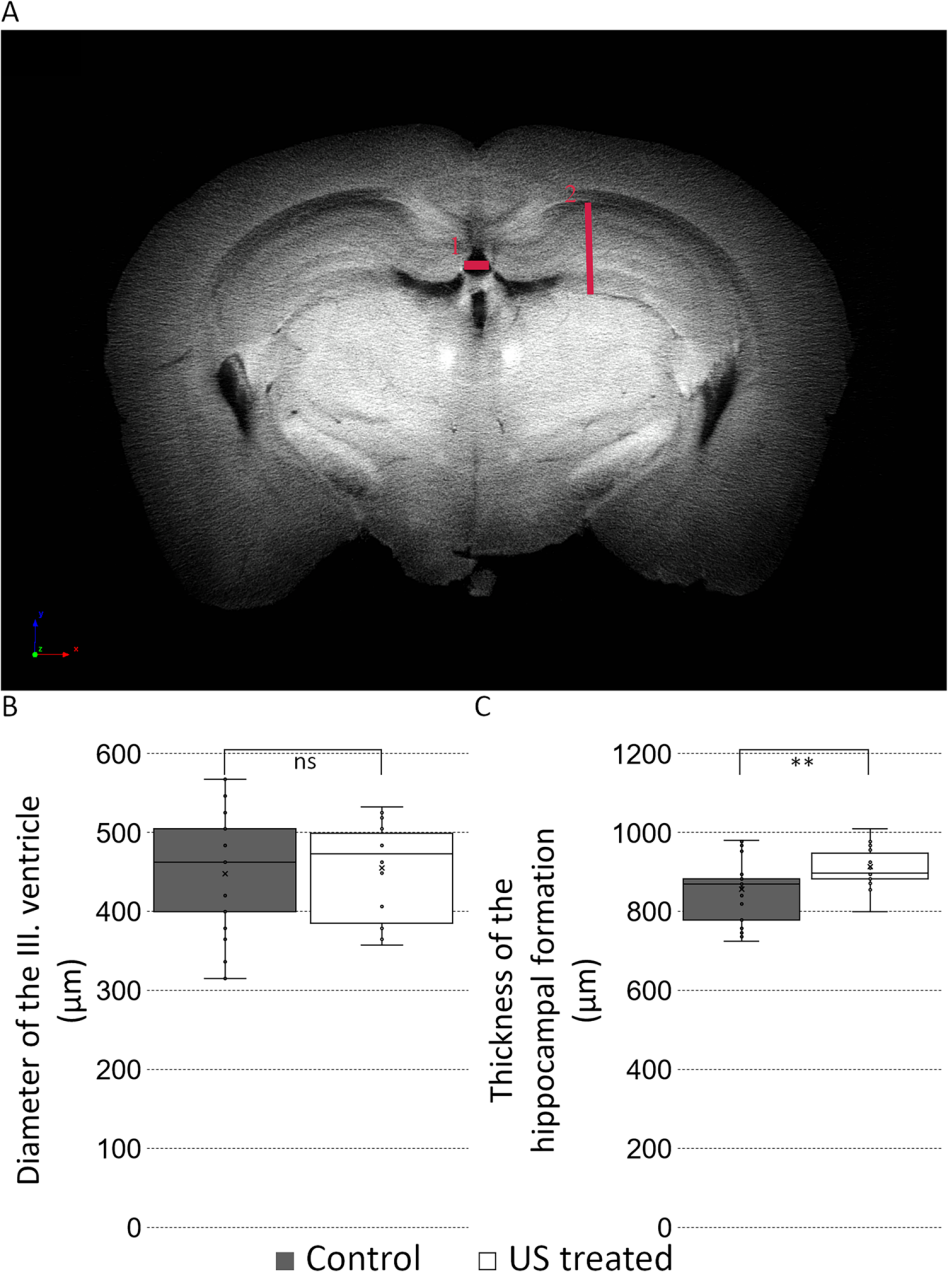
Repeated ultrasound prevents hippocampal atrophy. Quantification of the largest diameter (μm) of the III ventricle (localization of measurement 1. red line in (**A**) panel; numeric data in (**B**) panel, p = 0.84, control 449 ± 24.8980, US treated 456 ± 21.8350). The thickness of the hippocampal formation (μm) (localization of measurement 2. red line in (**A**) panel, numeric data in (**C**) panel: p = 0.01, control 861 ± 27.5193, US treated 912 ± 13.1125). Significant difference labeled by: **p ≤ 0.01. The MANOVA test showed a significant difference; p: 0.017. Scale bar: 800 µm.

The following parameters of the brains of one-year-old mice were examined with microCT: total whole brain volume and weight. On coronal brain sections at the level of the posterior commissure (as a well-visible anatomical landmark) the largest horizontal diameter of the III ventricle and the diameter of the short axis of the hippocampal formation were measured. All diameters were measured by three expert examiners independently. In the case of total brain volume and weight, we chose a significance level of p ≤ 0.01 (respectively to the dissection, which may cause injuries on the basal surface); while in every other case, it was p ≤ 0.017. We found a significant difference between the control and the US-treated group in the case of hippocampus formation thickness, the diameter was larger in the US-treated group (Figs. 6, 7).

## Discussion

The main limitations of the study arise from the animal model and the plasticity of the central nervous system since spine formation is a lifelong process. However, the control animals underwent the same procedure; the surgical intervention, electroporation, and stress may influence the susceptibility of neurons to ultrasound or may disturb the normal maturation of the central nervous system. The applied parameters represent the output of the transducer, and the peak negative acoustic pressure and average intensity of the US were calculated mathematically. The reconstructed neurons and dendrites are selected by experts in the field, however, it can be subjective.

Although the fine molecular and morphological changes of the CNS were not the focus of earlier investigations, it was confirmed that ultrasound examination in pregnancy has no adverse maternal or perinatal outcome and does not modify the normal physical or neurological development, furthermore does not increase the incidence of malignancies, subnormal intellectual performance or mental diseases in humans^18^. Consequently to the harmless effects of diagnostic US, in many countries, the obligatory US screening is performed several times until birth (on the 12, 18, and 32 weeks). Upon request, a US examination can be done also on the 28th and 38th weeks, as well as a “3D or 4D baby movie” at any time point of the pregnancy, with or without any medical recommendation^19^. To mimic the effect of US examination, we labeled the hippocampal CA1 neurons at 14.5 embryonic days in mice, which corresponds to the events of 11–12th gestational weeks in humans^20^, thus, the applied US exposures may hit the neurons in approximately the same maturation state in both species. US stimulation (6.7-MHz) for 30 min may disturb the proliferation of neuroprogenitors, disturb the migration of newborn neurons, and the lamination of the hippocampus and the neocortex. Of note, ultrasound exposure may cause a different response in neurons of the hippocampus and the neocortex, nevertheless, US exposure increases the number of dendrites in both CA1 and Layer 5 developing pyramidal cells in vivo^4,5,7,21^. Our previous study showed that a single scanning US exposure with the applied parameters led to an increase in the number of dendrites^4^. However, the other morphometric parameters of the dendritic tree remained unchanged on P3 L5 pyramidal cells. These cells showed a particularly similar result, specifically in terms of the elaborated dendritic tree, to the analyzed P28 CA1 pyramidal cells. The non-corresponding morphological properties could be attributed to immaturity (P3 vs P28 age) and the different types and developmental stages of neurons (L5 vs CA1 neurons). The US signaling has multiple pathways, including non selective cation channels (such as TRPA1, TRPC1) and direct physical effects, however a study indicates major role of TRPC1 receptor, and the overexpression of the TRPC1 results stronger stimulation, also important of note the expression patterns of this proteins rather heterogeneous between individual neurons, however the reason of the downregulation is not well understood. The US waves mechanically modify the cell membrane properties and ion channels with the consecutive elevation of intracellular Na+ and Ca2+ levels^3,9,22,23^. The elevation of intracellular cation level may trigger the synaptic transmission and electrical activity in neurons and as an indirect effect, changes the activity of neurotrophic factors (such as BDNF) causing the alteration of neural plasticity^24^. As described earlier by Papp et al.^4^ (using the same methodology and mouse strain) according to the qPCR test (from whole brain, same age), the RNA expression level of BDNF did not change between the samples, which indicates that the BDNF gene expression has not been modified by the US^4^. The repetitive diagnostic US exposure according to earlier findings causes elevated protein levels of BDNF and c-FOS in neurons^4^. Hatch et al. also found that the US prevents the age-associated loss of dendritic structure in CA1 cells, which is caused by the increased BDNF levels related to US exposure^5,25^. BDNF, as a key neurotrophin in CNS development, increases not only the dendritic arborization but also the dendritic spine density of CA1 pyramidal neurons and has a pivotal role in synapse formation and dendrite morphogenesis through the Ilk-mediated pathway^10,26^. According to our RNA-seq data, the upregulation of *Ilk* and *Mir-546* genes may take a part in the increased dendritic arborization and spine density on the basal dendrites, since they promote the synapse formation at the late stage of neuronal development^26–31^. Bhattacharya and colleagues described a strong link between impaired ILK pathway and reduced synaptic plasticity in a rat model^30^; the decrease in ILK activity could possibly be attributed to a lower ratio of BDNF to proBDNF^32^. Earlier data did not find a difference between the spine density of US-treated vs control CA1 neurons, however, in that case, the sonication protocol and the design of the study were different^5^. Spine formation is very dynamic in young animals during the first month of life; and only around 5% of all spines can be induced later, by learning or experiences^33^. The spines are the pivotal territory of the synapses on CA1 neurons, in certain neurological diseases (Parkinson’s, Huntington’s diseases, schizophrenia, and Alzheimer’s disease) the spine density decreases^14–16^. The higher dose (time: 20 min, 3.5 MHz, MI: 1.4, TI: 1.0) of US decreased the cognitive functions according to behavioral test, a low dose (time: 4 min, 3.5 MHz, MI: 0.1, TI: 0.1) of US exposure improved the cognitive functions of rats (US was applied on embryonic 6, 12, and 18 days)^34^. The same effect US was observed on mice pups (time: 10- 20 or 30 min, 3.5 MHz, 65 mW, ISPTP = 1 W/cm^2^, ISATA = 240 W/cm^2^), where high dose prenatal ultrasound (on embryonic 14.5 days) impaired the brain function in the adult mouse^35^. The repetitive US stimulation reduces cortical atrophy in humans (in AD disease)^36^, which is similar to our result in mice, the strongly upregulated FGF20 effects is mediated by FGFR1 receptor, which receptor has the strongest expression in the hippocampus; enhancing our results the rs12720208 SNP variant carrier humans have significantly increased gray matter volume than the other genetic variation of FGF20, indicating the neurotrophic activity of FGF20 on neurons, on the other hand the FGF20 through MAPK and PI3K/AKT signaling cascades, mediating neuroprotective effects in vitro^37,38^.

According to our findings and with the earlier results we may conclude that repetitive US exposure changes the spine and dendritic morphological properties of CA1 neurons, as well as modify the gene expression in the hippocampus.

## Methods

### Animals

Time-mated pregnant CD1 (ICR, Charles River, Germany) mice were used for in-utero electroporation. We subjected three pregnant mice, with a total of 18 electroporated embryos (12 survived), to US stimulation. Post-dissection, we sampled the brains that exhibited strong GFP labeling patterns. This was achieved by using cytoplasmatic GFP to label the entire cells, including the spines, which allowed for the evaluation of CA1 neurons. Based on the GFP labeling pattern, we selected three brains for morphometric analysis at P28. For control experiments, we used three pregnant mice with 17 electroporated embryos (13 survived) and selected three for morphometry based on the GFP labeling pattern. For RNAseq analysis, we chose an additional nine US-stimulated and nine control mice at P28. We also subjected eight control and eight US-stimulated one-year-old animals to CNS analysis with microCT. Throughout the study, the control group consisted of animals that underwent the same procedures as the US-treated animals but were not exposed to the actual US stimulus. We exclusively used female animals in both the treated and control groups. To determine the embryonic age, we observed the copulation plugs, which we referred to as embryonic day 0.5 (E0.5) of the zygotes. Animals were handled and housed according to the guidelines of the Animal Care Committee of the University of Debrecen, Debrecen, Hungary (Approval No.: 15/2020/DEMAB), and the national laws and regulations of the European Union (Directive 2010/63/EU).

### In-utero electroporation with EGFP plasmid

Neuronal morphological changes after US exposure were assessed on GFP-labeled CA1 pyramidal neurons. To reduce the inherent variability of pyramidal cell morphology, we compared cells born on the 14.5th embryonic day, migrated into the same layer, and thus were likely in a close differentiation state. We followed the previously described method^4^. Briefly, the electroporated plasmid DNA (coding fluorescent reporter genes) provides a stable gene expression only in those postmitotic neurons that leave the cell cycle at the same time as the electroporation. Pregnant mice at E14.5 were anesthetized deeply with sodium pentobarbital (50 mg/kg). The uterine horns were exposed through a median abdominal incision and the plasmid solution was injected into the right lateral ventricle of the embryos. The uterus, containing embryos, was then moistured with saline, placed back into the abdominal cavity, and closed by sutures. The injected plasmid solution (1 mg/ml) contained two vectors: (1) a cre-dependent GFP expression vector (pCALN-loxp-GFP-loxp-CS3, designed in our lab) and (2) a cre-recombinase expressing vector (pCAG-cre) was provided by Dr. J. Miyazaki (Division of Stem Cell Regulation Research, Osaka University Medical School, Osaka, Japan).

### Ultrasound exposure

Four days after in-utero electroporation (on E18.5) mice were deeply anesthetized with Na-pentobarbital (50 mg/kg), followed by a 10-min-long US stimulus. GE Logiq V2 ultrasound device was used at a constant frequency of 3 MHz, and mechanical and thermic indexes were both kept under 1.0 (MI = 0.9; TI = 0.8; other parameters: D: 17, DR: 69 dB, AO%: 100). The probe was 4C-RS convex transducer (frequency range: 2.0–5.0 MHz, number of elements: 128, convex radius: 60 mmR, FOV: 55°, footprint: 18.3 × 66.2 mm, B-mode imaging frequency: 2.0, 3.0, 4.0, 5.0 MHz, harmonic imaging frequency: 3.0, 4.0, 5.0 MHz). The calculated peak negative acoustic pressure was 1.557 MPa, and the average intensity during the exposition was 56 mW/cm^2^. The US head was covered by AquaUltra Basic US gel (Ultragel, Budapest, Hungary) and (in case of the in-utero US exposure) it was gently put on the abdomen of the pregnant animals or (during postnatal treatments) directly to the skull of the mice. The first in-utero stimulus was applied on E18.5, followed by four further stimuli after birth during the first four postnatal weeks (once a week). On E18.5 during the treatment, the focus was kept on the midline of the pregnant animals, to ensure that all the embryos got the same dose of US, and the transducer was in contact with the entire abdominal region of the pregnant animals. During postnatal treatments, unfocused ultrasound was applied to the small size of the skull. For microCT analysis, 8-8 animals (US treated vs control) lived for 12 months, later they were also sacrificed under deep anesthesia. These animals were transcardially perfused with 4% PFA followed by saline after perfusion brains were isolated from the skull.

### Immunohistochemistry

P28 mice were sacrificed by decapitation, and whole brains were carefully dissected and immersion fixed in 4% paraformaldehyde in 0.1 M phosphate-buffered saline (PBS, pH 7.4) overnight at 4 °C. Brain samples were then rinsed with PBS and embedded in 4% agarose, and 100 μm thick free-floating coronal sections were made with a vibratome (Leica VT 1000 S, Biosystems, Wetzlar, Germany). Sections were incubated in anti-GFP primary antibody (1:2000, ab13970, Abcam, Cambridge, UK) for 2 days at 4 °C followed by the incubation (overnight at 4 °C) with the fluorescent secondary antibody (anti-chicken Alexa Fluor-488, 1:500, Invitrogen, Carlsbad, CA, USA). All antibodies were diluted in PBS (pH 7.4) supplemented with 0.3 M NaCl and 0.3% Triton X-100. At the end of the protocol, sections were incubated with cell nucleus-specific DAPI (Sigma, D9542, 100 ng/ml) for 2 h at room temperature to help determine layer boundaries. Sections were mounted in Hydromount medium (National Diagnostics Atlanta, GA, USA), and confocal images were obtained with an Olympus FV3000 (Olympus Ltd., Tokyo, Japan) confocal microscope. After the fluorescent image collection, the immunofluorescent signal was converted into DAB-labeled samples (Suppl. 4). The coverslips were removed in PBS which was followed by incubating the sections overnight in donkey biotinylated anti-chicken IgG (1:200, Jackson ImmunoResearch Laboratories, PA, USA), and then with avidin–biotin complex (1:200, Vector Labs, CA, USA) for 2 h. The visualization was performed by DAB peroxidase substrate kit (Vector Labs, CA, USA) according to the supplier's protocol.

### Neuron reconstruction and morphometry

In the case of the sampling cells, the criteria were (1) the same layer in the labeled region, (2) strong GFP labeling, (3) sparse labeling, related to the reconstruction, and (4) the ability to follow the dendrites in the neighboring slices. Spine density analyses were performed on first-order branches of apical or basal dendrites, proximal to their branching points, with a diameter between 0.5 and 1.5 µm. Confocal images were collected at 0.5 μm steps with 40× (Olympus, UplanFLN, NA: 1.30) oil immersion objective. On immunofluorescent samples, the secondary branches of the apical and basal dendrites were traced and dendritic spines were labeled automatically using Imaris software (ver 9.5 Bitplane, Oxford Instruments, UK). Each automatic reconstruction was verified by an expert, manual correction happened e.g. if the software did not label spines, recognize dendritic crossing as a spine, counting the bifurcated spine as 2 spines. On the DAB-stained samples (Suppl. 3), we selected CA1 (17 control and 16 US treated) pyramidal cells for 3D reconstruction with Neurolucida software, the detailed description of the metrics used can be found in the manual of Neurolucida (ver 11.07, MBF Bioscience, Williston, VT, USA). The neuronal cell bodies were carefully drawn at all Z levels in the images, the neural dendrites were also particularly reconstructed, taking special note of process diameter. The morphometric parameters determined from the reconstructions were the total number of dendrites; highest order; average segment length; average segment tortuosity; average segment diameter; dendrite length; average terminal distance; mean length. With Imaris software, we analyzed the effects of repetitive US on the secondary branches of dendrites following spine characteristics: spine density; spine length; maximal, minimal, mean spine diameter, and spine volume. The person who did the analysis was blind in the case of the evaluation.

### Whole mouse brain sample preparation for contrast-enhanced computed tomography and morphological measurements

8–8 animals (5 times US treated vs control) surviving for 1 year, were sacrificed under deep anesthesia (Na-pentobarbital (50 mg/kg)). The animals were transcardially perfused with 4% PFA followed by saline, then brains were postfixed in 4% PFA at 4 °C overnight for 1 week. After two washing phases in PBS for 10–10 min, samples went through an ascending series of grade alcohol from 70% ethanol to dehydrate the brain tissue. For staining, we used 1% iodine dissolved in cc. ethanol with a 24-h incubation time at room temperature. To rehydrate our samples, we put them in a descending series of graded alcohol for 10 min each and finally rinsed them in distilled water. Iodine-stained whole brain samples were scanned with SkyScan 1272 compact desktop micro CT system, using the following scanning parameters: image pixel size: 3.5 μm; matrix size: 2688 × 4032 (rows × columns); source voltage: 70 kV; source current: 142 μA; rotation step (deg): 0.1, filter = Al 0.25 mm. Scan duration: 120 min. Reconstruction of the cross-sectional images from tomography projection images was performed with the SkyScan NRecon software (version 1.1.19). Post alignment, beam-hardening correction, ring artifact correction, and smoothing were completed during post-processing of the image data; the output formats were DICOM and .bmp images. The 3D Volume rendering tool was provided by Dataviewer software (version: 1.5.6.2.64-bit). On the coronal slices, we measured the longest latero-lateral diameter of the III ventricle (at the level of the posterior commissure as well as a visible landmark) and the diameter of the short axis of the hippocampal formation at the level of the uppermost point (the resolution of the micro CT is not able to detect the layers of the hippocampus). The total brain volume was calculated by Dataviewer software.

### Total RNA preparation and quality assessment for RNAseq analysis

One hour after the last US treatment, we sacrificed 9 control and 9 US-treated mice (P28) with Na-pentobarbital (150 mg/kg) for RNAseq analysis. After removing brains, we isolated the whole hippocampi from both sides, homogenized them, and immersed them in TRIzol (Ambion, Life Technologies, CA, USA). After RNA extraction, its concentration and A260/280 ratio were measured with a spectrophotometer (DeNovix, Inc., Wilmington, DE, USA), and the 9 samples from both US-treated and control groups were pooled (10–10 μg from each sample). Then the total RNA sample quality was checked on Agilent BioAnalyzer (Agilent Technologies, Inc., Santa Clara, CA, USA) using Eukaryotic Total RNA Nano Kit according to the manufacturer's protocol. Samples with an RNA integrity number (RIN) value of 7 were accepted for the library preparation process. To obtain global transcriptome data, a high throughput mRNA sequencing analysis was performed on the Illumina sequencing platform (Illumina, Inc., San Diego, CA, USA). RNA-Seq libraries were prepared from total RNA using Ultra II RNA Sample Prep kit (New England BioLabs) according to the manufacturer’s protocol. Briefly, poly-A RNAs were captured by oligo-dT conjugated magnetic beads then the mRNAs were eluted and fragmented at 94 Celsius degrees. First-strand cDNA was generated by random priming reverse transcription and after the second-strand synthesis step, a double-stranded cDNA was generated. After repairing ended, A-tailing and adapter ligation steps and adapter-ligated fragments were amplified in enrichment PCR and finally sequencing libraries were generated. Sequencing runs were executed on Illumina NextSeq 500 instrument using single-end 75 cycles sequencing. Raw sequencing data (fastq) was aligned to mouse reference genome version MM10 using the HISAT2 algorithm and BAM files were generated. Downstream analysis was performed using StrandNGS software (http://www.strand-ngs.com). BAM files were imported into the software, DESeq algorithm was used for normalization. We used Cytoscape (ver 3.10.1) software package for performing network and functional enrichment analysis followed by mapping of the GO Biological processes using EnrichmentMap (ver 3.3.6) app^17,39^.

### Statistical evaluation

The morphometric data between control and US-treated animals were compared using the Mann–Whitney *U* test or Two Sample *t*-test using OriginPro (OriginLab Corporation, Northampton, MA, USA) and PAST software (ver 4.14)^40^, the results of these analyses are shown on the box plots. Bonferonni correction made for a large number of statistical comparisons on small numbers of animals (3–3 animals each group) (p ≤ 0.05 changed to p ≤ 0.017). Significance levels indicated by: *p ≤ 0.017 or **p ≤ 0.01. Behind this, we also performed MANOVA analysis, between the morphometrics data with PAST software, results are indicated at the end of Figure legends (significance level: p ≤ 0.05). To analyze the result of RNA-seq *Z*-test with Benjamini–Hochberg FDR was used to determine differentially expressed genes between conditions. Boxes in the Figures indicate the middle 50% of the data with the median. Bars indicate the upper and lower 25–25% of data while the asterisk labels are the outlier data. The means are indicated as small crosses in the boxes.

### Ethics statement

The animal study was reviewed and approved by the Committee for Animal Research Studies at the University of Debrecen. The study is reported in accordance with ARRIVE guidelines.

### Supplementary Information

Supplementary Information 1.

Supplementary Video 1.

Supplementary Information 2.

Supplementary Information 3.

Supplementary Legends.

## Data Availability

The original contributions presented in the study are included in the article/supplementary material. The RNAseq raw data is available (http://www.ncbi.nlm.nih.gov/bioproject/PRJNA1058398), further inquiries can be directed to the corresponding author.
