# Supplementary figures and images for: Repeated diagnostic ultrasound exposure modifies the structural properties of CA1 dendrites and alters the hippocampal transcriptome
Source: Sci Rep. 2024 May 22;14:11713. doi: 10.1038/s41598-024-62621-y (PMC11111781; doi:10.1038/s41598-024-62621-y)

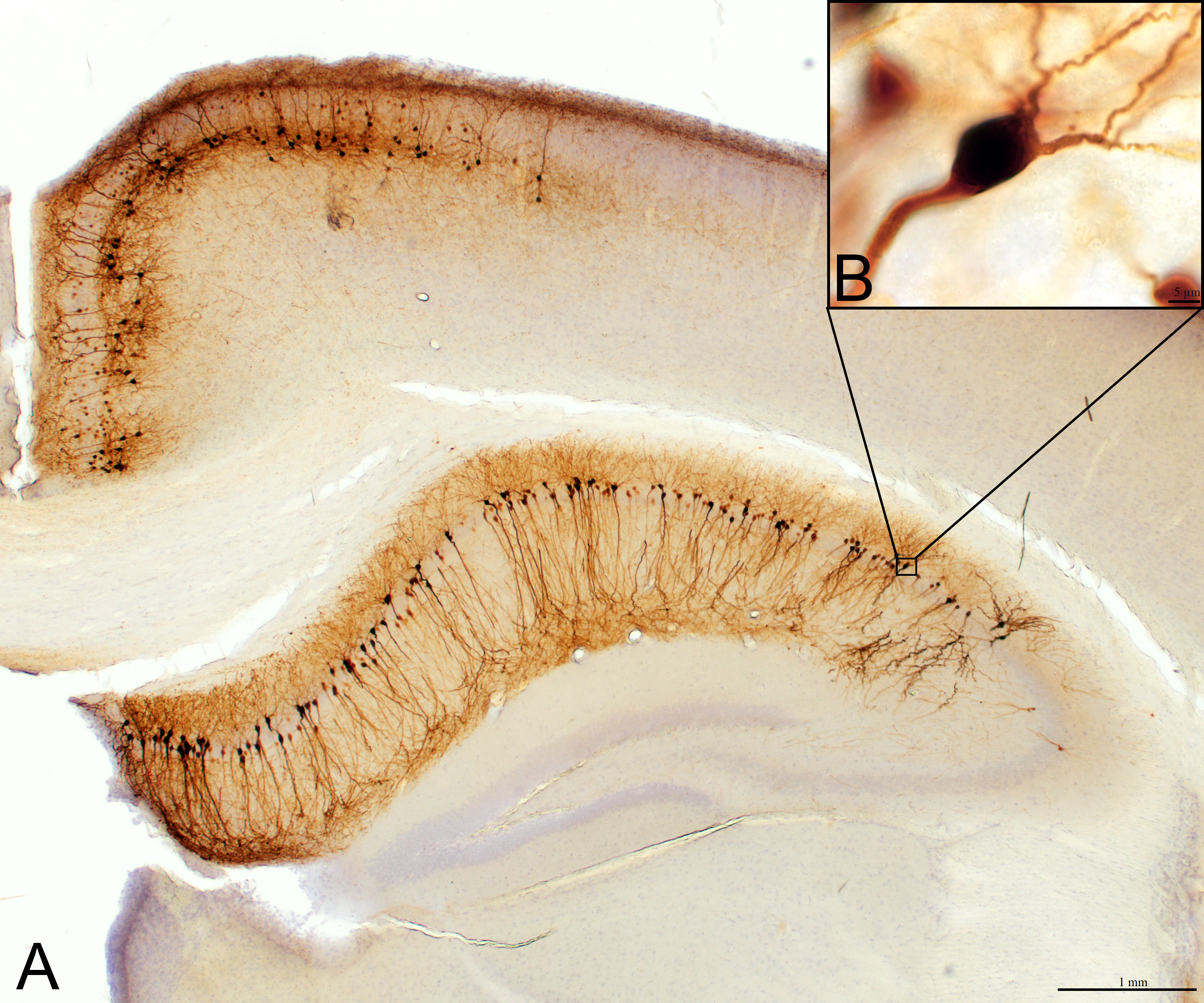

Supplement: Supplementary file 4 — Supplementary Information 3. [file 41598_2024_62621_MOESM4_ESM.tif]
