# Supplementary material for: Repeated diagnostic ultrasound exposure modifies the structural properties of CA1 dendrites and alters the hippocampal transcriptome
Source: Sci Rep. 2024 May 22;14:11713. doi: 10.1038/s41598-024-62621-y (PMC11111781; doi:10.1038/s41598-024-62621-y)
Supplement: Supplementary file 5 — Supplementary Legends. [file 41598_2024_62621_MOESM5_ESM.docx]

**Supplementery files figure legends**

Suppl. 1.

The changes in the expression profile of hippocampal transcriptome after repeated US exposure.

Suppl. 2.

Representative sample about the automatic reconstruction of a dendritic segment (yellow) and the labeled spines (blue).

Suppl. 3.

The raw data of the morphometry.

Suppl.4.

The cells were labeled with GFP, and the signal was enhanced with anti-GFP and visualized with diaminobenzidine. (A) Labeled pyramidal neurons were found in the CA1 region of the hippocampus as well as the cingulate and adjacent cortical area. (B) A CA1 pyramidal neuron is shown in high magnification to obtain the quality of GFP labeling for reconstruction. Scale bars: 1 mm (A) and 5 μm (B).
